# Influence of Latent Heating over the Asian and Western Pacific Monsoon Region on Sahel Summer Rainfall

**DOI:** 10.1038/s41598-017-07971-6

**Published:** 2017-08-09

**Authors:** Shan He, Song Yang, Zhenning Li

**Affiliations:** School of Atmospheric Sciences, Sun Yat-sen University, Guangzhou, Guangdong, China

## Abstract

There has been an interdecadal shift towards a less humid state in Sahel summer rainfall since the 1960s. The decreased Sahel summer rainfall was associated with enhanced summer latent heating over the South Asian and western Pacific summer monsoon region and anomalous zonal-vertical cell of the Asian summer monsoon circulation, indicating that the latent heating plays a significant role in the change in Sahel rainfall. The effects of the latent heating over different monsoon domains on the Sahel rainfall are investigated through several model experiments. Results show that the remote monsoon heating mainly affects Sahel rainfall by generating changes in the zonal-vertical atmospheric circulation.

## Introduction

The Sahel summer rainfall exerts a significant influence on both local ecology and local economy. It varies apparently from synoptic timescale to decadal timescale. A drying trend in the second half of the 20^th^ century under global warming appeared most obviously in Sahel in summer^1, 2^. The shift from a humid state in the 1950s and 1960s into a lesser one in the 1970s and 1980s with an abrupt change around 1969 has been confirmed^3–5^. Sahel droughts can also influence global climate by means of transporting dust^6^. The drying trend in Sahel is primarily related to global sea surface temperature (SST) variation^7, 8^, such as the Pacific Decadal Oscillation^1^, El Niño-Southern Oscillation and South Atlantic SST, which contributes to different rainfall variances through several mechanisms, for instance, the Pacific North America Oscillation^9–12^. The influence of SST on Sahel rainfall reduction can be attributed to both the internal variability and the externally forced change in SST^13^. Previous studies discovered several factors that influence the rainfall variation on various timescales. The Sahel rainfall is mainly associated with cloud clusters, which are modulated by several factors including the mid-tropospheric African easterly jet stream and the tropical easterly jet stream, among others^14^. Therefore, its variation is intimately correlated with the variations of the West African westerly jet stream and the African easterly waves^15, 16^, which indicate a teleconnection associated with rainfall. Besides, an increase in local albedo, which discloses desertification, can cause decreased precipitation^17–21^, and local vegetation interaction enhances rainfall variability^12, 22^. However, the effects of increased greenhouse gases and changes in anthropogenic aerosols vary among models, with some model results indicating drought and others but some indicating normal rainfall conditions^13, 23, 24^.

The Sahel is located in Afro-Eurasia, which is influenced by major summer heat sources^25, 26^ and monsoon systems with substantial latent heating^27, 28^. Since both mean diabatic heating and its fluctuation affect the mean climate^29^, changes over the Sahel region can be induced remotely by the Asian monsoon to the east. Furthermore, zonal-vertical cell of the Asian summer monsoon circulation associated with the diabatic heating gradient over Afro-Eurasia^26, 30^ and the Rossby wave response to the west of subtropical monsoon heating can influence the Sahel summer rainfall directly or affect it indirectly by modulating local meridional circulation^31, 32^. The Asian summer monsoon^33^ and Sahel summer rainfall as a whole are influenced by the same factors, and demonstrated to coexist as twin features of multi-scale forcing^34^. In short, there is a close connection between the Asian summer monsoon and Sahel summer rainfall. Hence, what is the role of the monsoon-desert mechanism in the Sahel rainfall reduction?

In this study, we analyze the NOAA’s Precipitation Reconstruction over Land and the NCEP/NCAR Reanalysis, and conduct numerical model simulations. These results demonstrate that the interdecadal change in the Asian summer monsoon plays an important role in the change in Sahel summer rainfall.

## Results

### Observed change in rainfall and its accompanied features

**Figure 1:**
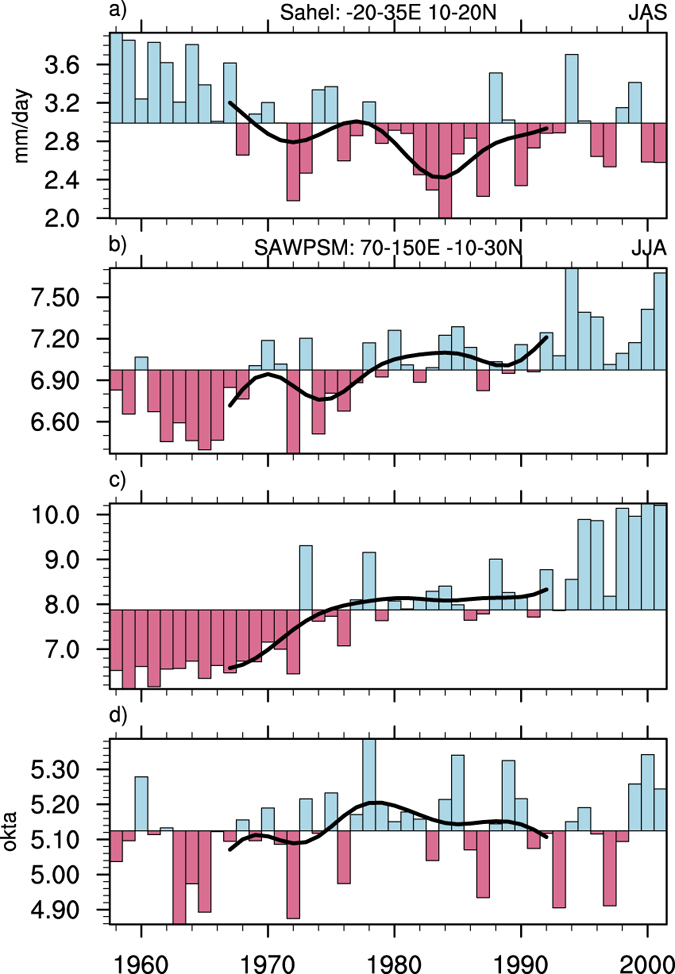
Time series of JAS Sahel rainfall, JJA SAWPSM precipitation and JJA SAWPSM cloudiness. Time series of (**a**) JAS rainfall over the Sahel in the PRECL precipitation data, (**b**) JJA SAWPSM precipitation in the NCEP Reanalysis data, (**c**) JJA SAWPSM precipitation in the ERA-40, and (**d**) JJA SAWPSM cloudiness (bar), and the weighted running average with 9-year Lanczos low-pass filter of the time series (solid line; see Methods). This figure was generated by the NCAR Command Language (Version 6.3.0) [Software]. (2016). Boulder, Colorado: UCAR/NCAR/CISL/TDD. http://dx.doi.org/10.5065/D6WD3XH5.

The Sahel is a narrow semi-arid region with precipitation mainly during summer (from July to September), ranging up to over 14 mm/day (Supplementary Fig. S1). The driest years of Sahel were the early 1980s. The difference between 1960–1969 and 1980–1989 indicates that summer rainfall decreased considerably over Sahel (by 20.5%), and increased slightly to the south (Fig. 1a and Supplementary Fig. S2a). The observed change in divergent/convergent wind shows anomalous ascents over southern Asia and the western Pacific and anomalous descents over Sahel (Supplementary Fig. S3). Meanwhile, an interdecadal change (by a 5.2% increase in the NCEP/NCAR Reanalysis and 23.3% in the ERA-40) in precipitation occurred in the South Asian and western Pacific summer monsoon (SAWPSM) region between 1960–1969 and 1980–1989 (Fig. 1b and c), suggesting a remote latent heating modulation of Sahel rainfall and an anti-correlation between the two during 1960–1989. The change in precipitation mainly emerged in the western Pacific and around the Strait of Malacha, with a slight decrease near the equator (Supplementary Fig. S2b and c). The increase in cloudiness also suggests an increase in precipitation in the SAWPSM region (Fig. 1d). In addition, the pattern of precipitation change over the land of Asia and Africa shows coherent variability (Supplementary Fig. S2a) ^35, 36^. However, what we attempt to focus on in this study is the role of the monsoon heating over the SAWPSM region^31^, which includes vast tropical-subtropical oceans with considerable diabatic heating (Supplementary Fig. S5). When oceans are included in the model, changes in Sahel rainfall and SAWPSM precipitation could be out of phase^36^.

This increase in precipitation is probably linked to the increase in the northern Indian Ocean and western Pacific SST (Supplementary Fig. S4) ^37^, as well as the increase in heating, while El Niño-Southern Oscillation and tropical Atlantic SST contribute to the high-frequency variation of Sahel rainfall^10–12^. However, both the precipitations over Sahel and that in the SAWPSM region have increased since the 1990s (Fig. 1). Because of the much missing data over tropical oceans, this decrease in cloudiness since the 1980s indeed indicated an opposite trend of precipitation over the subtropical oceans, although the whole SAWPSM region is included in consideration.

### Experiments with SAWPSM latent heating anomalies

To explore the possible influence of the decadal change in SAWPSM latent heating on Sahel summer rainfall, we carried out several experiments with the NCAR Community Earth System Model by modifying the heating over the SAWPSM region in several sensitivity tests (see Methods and Table 1).

**Table 1:** List of model experiments and experiment designs.

	Region applied	Heating coefficient	Integration (year)	Spin up (year)
CON CON_60 CON_80	None	\	18	3
SEN_A	70°E-150°E, 10°S-30°N	1.1		
SEN_W	70°E-110°E, 10°S-30°N	1.1		
SEN_E	110°E-150°E, 0°-20°N	1.1		
SEN_COOL1	70°E-150°E, 10°S-30°N	0.8		
SEN_COOL2	20°W-35°E, 10°N-20°N	0.8		

**Figure 2:**
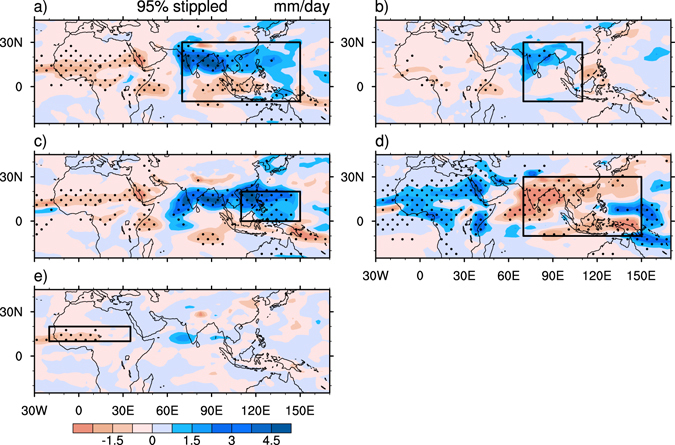
Differences in JAS precipitation. (**a**) Between SEN_A and CON, (**b**) between SEN_W and CON, (**c**) between SEN_E and CON, (**d**) between SEN_COOL1 and CON, and (**e**) between SEN_COOL2 and CON. The boxes define the anomalous heating regions for sensitivity experiments. The stippled area denotes the region where the change significantly exceeds the 95% confidence level (Student’s *t*-test). This figure was generated by the NCAR Command Language (Version 6.3.0) [Software]. (2016). Boulder, Colorado: UCAR/NCAR/CISL/TDD. http://dx.doi.org/10.5065/D6WD3XH5.

**Figure 3:**
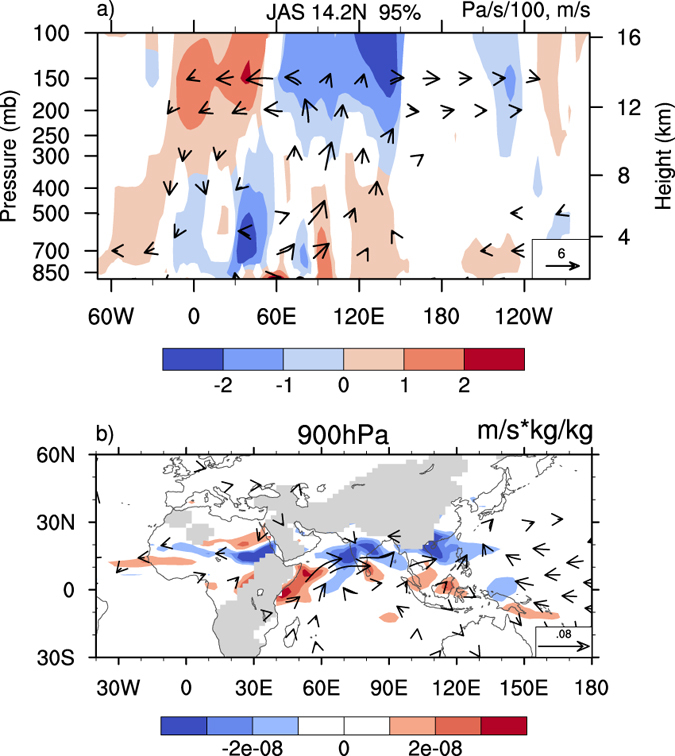
Differences in JAS omega, zonal wind, meridional wind, and water vapor flux. Differences in JAS (**a**) omega, zonal wind (vector) and meridional wind (shading) at 14.2°N between SEN_A and CON, and (**b**) water vapor flux (vector) and its divergence (shading) at 900 hPa between SEN_E and CON. The meridional wind and vectors denote the regions where the changes are statistically significant at the 95% confidence level according to the *t*-test. Omega is scaled so that the vertical motion is visible. This figure was generated by the NCAR Command Language (Version 6.3.0) [Software]. (2016). Boulder, Colorado: UCAR/NCAR/CISL/TDD. http://dx.doi.org/10.5065/D6WD3XH5.

The first experiment, referred to as experiment SEN_A, was performed to identify the influence of heating anomaly over the whole SAWPSM region. In this experiment (Fig. 2a), precipitation increases substantially over the northern SAWPSM region but decreases slightly over the southern SAWPSM, indicating that precipitation mainly increases associated with increasing SAWPSM latent heating. The local responses to the heating anomaly in the model match the general features of observed precipitation anomaly. The precipitation over the Sahel region decreases with enhanced monsoon heating, a feature consistent with the observed (Fig. 1a). There exist anomalous rising motion over the SAWPSM region, where anomalous moist convective heating is balanced by the adiabatic cooling associated with the ascent^38^, and anomalous sinking motion over the Sahel region (Fig. 3a). The results indicate an enhancement of the zonal-vertical cell of Asian summer monsoon circulation that links the Sahel and tropical Asia^30^. There are anomalous southerlies below the maximum rising motion over the SAWPSM region and anomalous northerlies below the maximum sinking motion over Sahel. The anomalous northerlies were caused by the vortex shrinking associated with the SAWPSM-induced sinking motion, in agreement with the Sverdrup relation (see Methods)^31^. Thus, while more water vapor is transported from the Indian Ocean and the South China Sea to maintain stronger Asian monsoon, the Sahel region receives less water vapor supply from the Gulf of Guinea.

We conducted additional experiments by increasing the heating of deep convection over Southeast Asia and the western North Pacific, respectively (Supplementary Fig. S5), referred to as experiments SEN_W and SEN_E, to identify the atmospheric responses over surrounding regions to these heating anomalies. Since the summer monsoons over these two regions, with different life cycles, are characterized by different moisture influxes and modes with different symmetric and asymmetric components^39^, they can be investigated separately. Moreover, we decreased the heating over the SAWPSM region, referred to as experiment SEN_COOL1, and decreased the heating over the Sahel region, referred to as experiment SEN_COOL2. Experiment SEN_COOL1 was conducted to clarify the linear atmospheric response to decreased SAWPSM heating, which was opposite to the response to increased SAWPSM heating, while experiment SEN_COOL2 was aimed to discern the feedback effect of decreased Sahel rainfall. In experiment SEN_W (Fig. 2b), precipitation increases over South Asia and decreases over the Sahel region with anomalous atmospheric circulation similar to that in experiment SEN_A. In experiment SEN_E (Figs 2c and 3b), precipitation increases over the SAWPSM region and decreases over the Sahel region with anomalous vertical motion similar to that in experiment SEN_A. An enhancement of the western North Pacific monsoon heating is accompanied by an increase in South Asian monsoon precipitation, which overlaps a cyclonic anomaly in the lower levels of the atmosphere (Fig. 3b). The cyclonic anomaly may be caused by the increasing heating to its east, in agreement with a Gill-type response^40^.

In experiment SEN_COOL1 (Fig. 2d), precipitation increases over the Sahel region, resulted from the precipitation reduction over the SAWPSM region. Within this limit, the Sahel summer rainfall changes monotonously with the precipitation change over the SAWPSM region (Fig. 2a,d). In experiment SEN_COOL2 (Fig. 2e), a decrease in Sahel rainfall results in a slight increase in precipitation over the SAWPSM region through the enhancement of the thermally-driven zonally asymmetric circulation, which is a positive feedback between the precipitation over the Sahel region and the precipitation over the SAWPSM region. In other words, the enhanced SAWPSM monsoon heating decreases Sahel rainfall (Fig. 2c), and the decreased rainfall and local latent heating in return increase the originally enhanced SAWPSM precipitation slightly.

In addition, experiments CON_60 and CON_80 were performed to distinguish whether the SST changes are a main cause of the SAWPSM heat anomaly. Despite some differences between the results from model and observation, a comparison with experiment CON_60 indicates that the precipitation in experiment CON_80 increases substantially over the western Pacific and the Bay of Bengal, instead of the Strait of Malacha, and decreases slightly near the equator (Supplementary Fig. S6). The model responses to the SST anomalies match the general features of observed precipitation anomaly. The results from experiments CON_60 and CON_80 imply that the SST change can be a main cause of the SAWPSM heat anomaly^37^. However, further research is needed to reveal the responsible mechanism.

**Figure 4:**
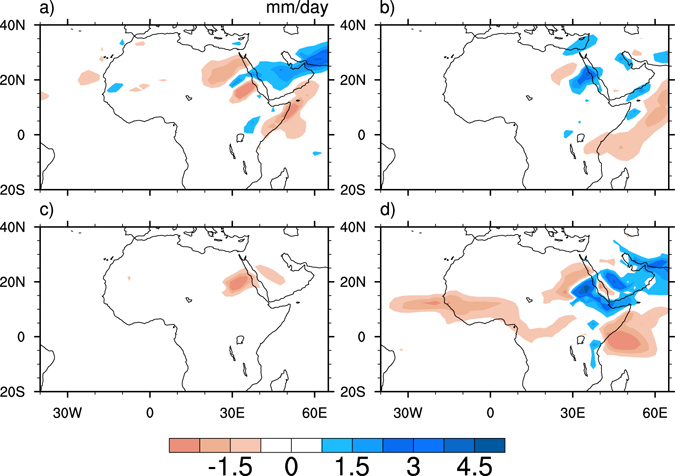
Differences in JAS moisture convergence. (**a**) $-\overrightarrow{{V}_{c}}\cdot {\rm{\nabla }}(\delta q)$, (**b**) $-(\delta \overrightarrow{V})\cdot {\rm{\nabla }}{q}_{c}$, (**c**) $-(\delta {q}_{c})$ ${\rm{\nabla }}\cdot \overrightarrow{{V}_{c}}$, and (**d**) $-{q}_{c}\,{\rm{\nabla }}\cdot (\delta \overrightarrow{V})$ between SEN_A and CON. This figure was generated by the NCAR Command Language (Version 6.3.0) [Software]. (2016). Boulder, Colorado: UCAR/NCAR/CISL/TDD. http://dx.doi.org/10.5065/D6WD3XH5.

Although the summer monsoons over Southeast Asia and the western North Pacific with different life cycles are characterized by different modes, increasing monsoon heating over both regions results in a decrease in Sahel summer rainfall. Anomalous sinking motion overlaps the decreasing Sahel rainfall, indicating that the dynamic process plays an important role in the rainfall reduction. Furthermore, moisture budget was computed and diagnosed (see Methods). Drought is mainly caused by the change in wind divergence with fixed moisture, and the influence of latent heating over the SAWPSM region on Sahel summer rainfall is mainly dynamical, with a smaller thermodynamic effect (Fig. 4). These model results are consistent with observational features.

## Discussion

The Sahel region has suffered from deficit summer rainfall since the 1960s and the rainfall variations from seasonal to interdecadal scales have been widely studied. It is also noticed that an interdecadal change in SAWPSM latent heating occurred together with the change in precipitation (Fig. 1). In this study, close attention is paid to the role of the monsoon-desert mechanism in Sahel rainfall reduction. Meanwhile, there exists an enhancement of zonal-vertical cell of the Asian summer monsoon circulation linking the Sahel region (Supplementary Fig. S3) and there is a negative correlation between the Asian heating and the Sahel rainfall on interdecadal scale (with a correlation coefficient of −0.58, which significantly exceeds the 99% confidence level). On the contrary, the increased heating does not inhibit the rainfall variability on interannual scale (with a correlation coefficient of 0.22, significant at the 90% confidence level).

Absence of negative correlation between the two since the 1990s is another interesting feature (Fig. 1). Meanwhile, the Atlantic multidecadal oscillation switched to its warm phase in the early 1990s, and the warm SST may weaken the longitudinal heating gradient over Afro-Eurasia. Then, a weakening occurs in the thermally-driven direct zonal-vertical cell of the Asian summer monsoon circulation^30^ and in the Rossby wave propagation from Asia to the Atlantic along anomalous westerlies. In consequence, the monsoon-desert coupling declines. However, a warm Atlantic multidecadal oscillation phase itself can generate anomalous westerlies with moist air to increase the Sahel rainfall^41^. As for the question of which is the dominant mechanism, further investigation is needed.

Results of several numerical experiments designed to examine this phenomenon also showed that the increasing heating over different parts of the SAWPSM region exerts a remote dynamic effect on the Sahel rainfall. The Asian monsoon conveys signals to the west through two mechanisms. One is the Rossby wave propagation to the west, which is induced by diabetic heating^31^ and produces sinking motion over Africa. The other is the transverse circulation modulated by the longitudinal heating gradient over Afro-Eurasia (Fig. 3a)^30^. The results of numerical experiments in this study support the hypothesis of the two mechanisms stated above. The role of the monsoon-desert mechanism in the Sahel rainfall reduction is thus confirmed. Further research is proposed to explore its role in the dominant influence of oceans. For experiment SEN_COOL2, a change in convection over the Sahel affects the Asian monsoon by enhancing the longitudinal heating gradient. Then, reinforcement occurs in the transverse Asian summer monsoon circulation, along with SAWPSM precipitation.

Although it may not be optimal to multiply the predicted heating of deep convection by an invariant coefficient, this should be sufficient to confirm the linkage between Sahel rainfall and SAWPSM precipitation. The fact that the local responses to the heating anomaly match the features of observed precipitation anomalies also supports the sufficiency (Fig. 2 and Supplementary Fig. S2). To replace the predicted heating in the model with a model-derived latent heating climatology is suggested for further investigation. Coupling experiments are also proposed to investigate the role of air–sea interaction induced by the heating anomaly over the SAWPSM region in the future, as the along-shore anomalous northerlies over the Sahel region induced by the heating anomaly (Fig. 3a) may result in offshore Ekman transport and upwelling, which can lead to cold SSTs. Hence, the intertropical convergence zone may shift to the south^42^ with less Sahel summer rainfall. In addition, the causes of increased heating and its relationship with the warming of northern Indian Ocean need to be studied in the future.

## Methods

### Observational data sets

The data sets used are as follows: (1) PRECL precipitation data provided by the NOAA/OAR/ESRL PSD, Boulder, Colorado, USA, from their web site at http://www.esrl.noaa.gov/psd/, which are on 0.5° × 0.5° grid from 1958 to 2001^43^; (2) monthly mean cloudiness on 2° × 2° grid from 1958 to 2001 from the ICOADS 2-degree Enhanced data provided by the NOAA/OAR/ESRL PSD, Boulder, Colorado, USA, from their web site at http://www.esrl.noaa.gov/psd/ ^44^; (3) convective and large-scale precipitation from the ERA-40 data set on 1° × 1° grid from 1958 to 2001^45^; (4) monthly mean SST from the Hadley Centre Sea Ice and Sea Surface Temperature data set on 1° × 1° grid from 1958 to 2001^46^; (5) monthly mean U wind, V wind and precipitation rate on 2.5° × 2.5° grid from 1958 to 2001 from the NCEP Reanalysis data provided by the NOAA/OAR/ESRL PSD, Boulder, Colorado, USA, from their web site at http://www.esrl.noaa.gov/psd/ ^47^; and (6) monthly mean outgoing longwave radiation on 1° × 1° grid from 1979 to 2001 from the Interpolated OLR data provided by the NOAA/OAR/ESRL PSD, Boulder, Colorado, USA, from their web site at http://www.esrl.noaa.gov/psd/.

### Lanczos filter weights

Calculates Lanczos filter weights^48^. The total number of weights is 19. The cut-off frequency of the ideal low-pass filter is 1/9. The power of the sigma factor is 1.

### CESM experiments

The Community Earth System Model Version 1.2.2 (CESM 1.2.2) was employed^49^. The component set of F_2000_CAM5 was chosen with the Community Atmospheric Model version 5 (CAM5) on 1.9° × 2.5° grid and 26 levels. Experiments were prescribed with monthly climatology of SST and sea ice from 1950 to 2010, and other external forcing such as CO_2_, ozone and aerosol with their values set at those in 2000. Each model integration is 18 years from the first day of the first year, and the mean values of the last 15 years are analyzed. Predicted heating of deep convection was multiplied by an invariant coefficient over different parts of the SAWPSM region in several sensitivity experiments from June to September, when the predicted heating of deep convection in the model was positive for each air parcel at each integration step. The specific coefficient is chosen according to the interdecadal change in the Sahel or SAWPSM precipitation (Table 1). In addition, two experiments CON_60 and CON_80 were conducted. All the setting of experiments CON_60 and CON_80 is the same as that in experiment CON, but the monthly climatology of SST and sea ice differ: for experiment CON_60, the period is from 1960 to 1969, whereas for experiment CON_80, it is from 1980 to 1989.

### Sverdrup relation

Also called Sverdrup balance^31^, it can be derived from the linearized barotropic vorticity equation for steady motion away from the equator:

1 $$\beta {v}_{g}=f\frac{\partial w}{\partial z}$$

where *v*_*g*_ is the geostrophic interior y-component (northward) and *w* is the z-component (upward) of the velocity. The equation suggests that when a vertical column of air is squashed, it moves toward the equator; and when it is stretched, it moves toward the pole.

### Diagnostic computation of moisture budget

In an equilibrium state^50^, moisture budget can be diagnosed with the following equation:

2 $$\begin{array}{rcl}\delta \bar{P} & = & -\delta \int \frac{\overline{\partial q}}{\partial t}+\delta \bar{E}-\int [\overrightarrow{v}\cdot \nabla (\delta q)]-\int [(\delta \overrightarrow{v})\cdot \nabla \bar{q}]-\int [(\delta q)\nabla \cdot \bar{\overrightarrow{v}}]\\  &  & -\int [\bar{q}\nabla \cdot (\delta \overrightarrow{v})]-\delta \int (\nabla \cdot \bar{{\overrightarrow{v}}^{^{\prime} }{q}^{^{\prime} }})\cdot +{\rm{higher}}\,{\rm{order}}\,{\rm{terms}}\end{array}$$

where ∫*X* indicates a vertical integral of *X* over the entire atmospheric column. *δX*, $\bar{X}$, and *X*′, respectively, represent the difference between the mean of the post-1965 period and that of the pre-1965 period, the mean of the pre-1965 period and the departure from monthly mean of *X*, each using monthly mean data sets. The tendency term of specific humidity is small and therefore neglected. The difference in precipitation is balanced mainly by moisture convergence or divergence. The total moisture flux convergence can be decomposed into two terms, in which the sub-monthly term $(\delta \int (\nabla \cdot \bar{\mathop{v}\limits^{\to ^{\prime} }{q}^{^{\prime} }}))$ is much smaller. Then, it can be decomposed into five terms, including the effect of the change in moisture gradient when total advective (rotational plus divergent) wind field is fixed, the contribution from the change in total wind field with a fixed moisture gradient, the effect of the change in domain-averaged moisture with a fixed divergent wind, the effect of the change in wind divergence with fixed moisture, and the higher order terms including the quadratic terms of “*dXdY*” type and the contribution from interannual variability, which can be conveniently ignored as it is small. The first two represent the effects of moisture convergence due to advection across the moisture gradient; the next two represent the effects of wind divergence/convergence.

### Graphic software

All figures were generated by the NCAR Command Language (Version 6.3.0) [Software]. (2016). Boulder, Colorado: UCAR/NCAR/CISL/TDD. http://dx.doi.org/10.5065/D6WD3XH5.

## Electronic supplementary material

Supplementary Information

## References

[1] Ma ZG, Fu CB (2007). Global aridification in the second half of the 20th century and its relationship to large-scale climate background. Sci. China Ser. D Earth Sci..

[2] Nicholson SE (1980). The nature of rainfall fluctuations in subtropical West Africa (Guinea Sahel Soudan). Monthly Weather Review.

[3] L’Hôte Y, Mahé G, Somé B, Triboulet JP (2002). Analysis of a Sahelian annual rainfall index from 1896 to 2000; the drought continues. Hydrological Sciences Journal.

[4] Ozer P, Erpicum M, Demarée G, Vandiepenbeeck M (2003). The Sahelian drought may have ended during the 1990s. Hydrological Sciences Journal.

[5] Greene, A. M., Giannini, A. & Zebiak, S. E. Drought return times in the Sahel: A question of attribution. *Geophys. Res. Lett*. **36** (2009).

[6] Prospero JM, Lamb PJ (2003). African Droughts and Dust Transport to the Caribbean: Climate Change Implications. Science.

[7] Palmer TN (1986). Influence of the Atlantic, Pacific and Indian Oceans on sahel rainfall. Nature.

[8] Folland CK, Palmer TN, Parker DE (1986). Sahel rainfall and worldwide sea temperatures, 1901–85. Nature.

[9] Hunt BG (2000). Natural climatic variability and Sahelian rainfall trends. Global Planet. Change.

[10] Camberlin P, Janicot S, Poccard I (2001). Seasonality and atmospheric dynamics of the teleconnection between African rainfall and tropical sea-surface temperature: Atlantic vs. ENSO. Int. J. Climatol..

[11] Ward MN (1998). Diagnosis and short-lead time prediction of summer rainfall in tropical North Africa at interannual and multidecadal timescales. Journal of Climate.

[12] Giannini A, Saravanan R, Chang P (2003). Oceanic Forcing of Sahel Rainfall on Interannual to Interdecadal Time Scales. Science.

[13] Held IM, Delworth TL, Lu J, Findell KL, Knutson TR (2005). Simulation of Sahel drought in the 20th and 21st centuries. Proc. Natl. Acad. Sci. USA.

[14] Nicholson SE, Grist JP (2003). The seasonal evolution of the atmospheric circulation over West Africa and equatorial Africa. Journal of Climate.

[15] Pu B, Cook KH (2012). Role of the west African westerly jet in sahel rainfall variations. Journal of Climate.

[16] Skinner CB, Diffenbaugh NS (2014). Projected changes in African easterly wave intensity and track in response to greenhouse forcing. Proc. Natl. Acad. Sci. USA.

[17] Charney J, Quirk WJ, Chow S-H, Kornfield J (1977). Comparative Study of The Effects of Albedo Change On Drought in Semi-Arid Regions. J. Atmos. Sci..

[18] Charney J, Stone PH, Quirk WJ (1975). Drought in the Sahara: A biogeophysical feedback mechanism. Science.

[19] Tucker CJ, Dregne HE, Newcomb WW (1991). Expansion and contraction of the Sahara desert from 1980 to 1990. Science.

[20] Courel MF, Kandel RS, Rasool SI (1984). Surface albedo and the sahel drought. Nature.

[21] Yongkang X, Shukla J (1993). The influence of land surface properties on Sahel climate. Part I: desertification. Journal of Climate.

[22] Zeng N, Neelin JD, Lau KM, Tucker CJ (1999). Enhancement of interdecadal climate variability in the Sahel by vegetation interaction. Science.

[23] Biasutti, M. & Giannini, A. Robust Sahel drying in response to late 20th century forcings. *Geophys. Res. Lett*. **33** (2006).

[24] Dong B, Sutton R (2015). Dominant role of greenhouse-gas forcing in the recovery of Sahel rainfall. Nat. Clim. Change.

[25] Yanai M, Tomita T (1998). Seasonal and interannual variability of atmospheric heat sources and moisture sinks as determined from NCEP-NCAR reanalysis. Journal of Climate.

[26] Li W, Chineke TC, Xin L, Wu G (2001). Atmospheric Diabatic Heating and Summertime Circulation in Asia-Africa Area. Adv. Atmos. Sci..

[27] Webster PJ (1998). Monsoons: processes, predictability, and the prospects for prediction. J. Geophys. Res. C Oceans.

[28] Webster PJ, Song Y (1992). Monsoon and ENSO: selectively interactive systems. Quarterly Journal - Royal Meteorological Society.

[29] Sardeshmukh PD, Sura P (2007). Multiscale impacts of variable heating in climate. Journal of Climate.

[30] Song Y, Webster PJ, Min D (1992). Longitudinal heating gradient: Another possible factor influencing the intensity of the Asian summer monsoon circulation. Adv. Atmos. Sci..

[31] Rodwell MJ, Hoskins BJ (2001). Subtropical anticyclones and summer monsoons. Journal of Climate.

[32] Liu P, Wu G, Sun S (2001). Local Meridional Circulation and Deserts. Adv. Atmos. Sci..

[33] Yang S, Lau KM (1998). Influences of sea surface temperature and ground wetness on Asian summer monsoon. Journal of Climate.

[34] Wu GX (2009). Multi-scale forcing and the formation of subtropical desert and monsoon. Ann. Geophys..

[35] Wang PX (2014). The global monsoon across timescales: Coherent variability of regional monsoons. Climate of the Past.

[36] Feudale L, Kucharski F (2013). A common mode of variability of African and Indian monsoon rainfall at decadal timescale. Clim. Dyn..

[37] Xie SP (2010). Global warming pattern formation: Sea surface temperature and rainfall. Journal of Climate.

[38] Rodwell MJ, Hoskins BJ (1996). Monsoons and the dynamics of deserts. Q. J. R. Meteorol. Soc..

[39] Murakami T, Matsumoto J, Yalagai A (1999). Similarities as well as differences between summer monsoons over Southeast Asia and the western North Pacific. J. Meteorol. Soc. Jpn..

[40] Gill AE (1980). Some simple solutions for heat‐induced tropical circulation. Q. J. R. Meteorol. Soc..

[41] Knight, J. R., Folland, C. K. & Scaife, A. A. Climate impacts of the Atlantic multidecadal oscillation. *Geophys. Res. Lett*. **33** (2006).

[42] Philander SGH (1996). Why the ITCZ is mostly north of the equator. Journal of Climate.

[43] Chen M, Xie P, Janowiak JE (2002). Global land precipitation: A 50-yr monthly analysis based on gauge observations. Journal of Hydrometeorology.

[44] Freeman E (2017). ICOADS Release 3.0: a major update to the historical marine climate record. Int. J. Climatol..

[45] Kållberg, P. W., Simmons, A., Uppala, S. & Fuentes, M. In *ERA-40 Project Report Series* 31 (ECMWF, Shinfield Park, Reading, 2004).

[46] Rayner NA (2003). Global analyses of sea surface temperature, sea ice, and night marine air temperature since the late nineteenth century. Journal of Geophysical Research D: Atmospheres.

[47] Kalnay E (1996). The NCEP/NCAR 40-year reanalysis project. Bulletin of the American Meteorological Society.

[48] Duchon CE (1979). Lanczos Filtering in One and Two Dimensions. Journal of applied meteorology.

[49] Kay JE (2015). The community earth system model (CESM) large ensemble project: A community resource for studying climate change in the presence of internal climate variability. Bulletin of the American Meteorological Society.

[50] Huang HP, Seager R, Kushnir Y (2005). The 1976/77 transition in precipitation over the Americas and the influence of tropical sea surface temperature. Clim. Dyn..

