# Supplementary material for: Influence of Latent Heating over the Asian and Western Pacific Monsoon Region on Sahel Summer Rainfall
Source: Sci Rep. 2017 Aug 9;7:7680. doi: 10.1038/s41598-017-07971-6 (PMC5550481; doi:10.1038/s41598-017-07971-6)
Supplement: Supplementary file 1 — Supplementary Information [file 41598_2017_7971_MOESM1_ESM.doc]

**Influence of Latent Heating over the Asian and Western Pacific Monsoon Region on Sahel Summer Rainfall**

Shan He, Song Yang & Zhenning Li

**Supplementary Figure S1**


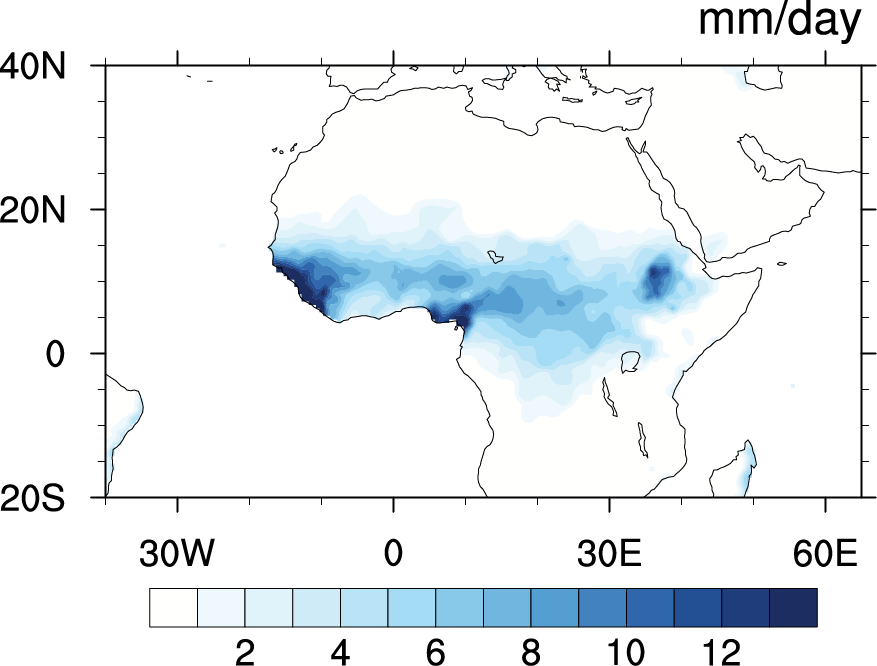


**Supplementary Figure S1 | Climatology of Sahel JAS rainfall.** Climatology of African JAS rainfall for 1950-1981. This figure was generated by the NCAR Command Language (Version 6.3.0) [Software]. (2016). Boulder, Colorado: UCAR/NCAR/CISL/TDD. <http://dx.doi.org/10.5065/D6WD3XH5>.

**Supplementary Figure S2**


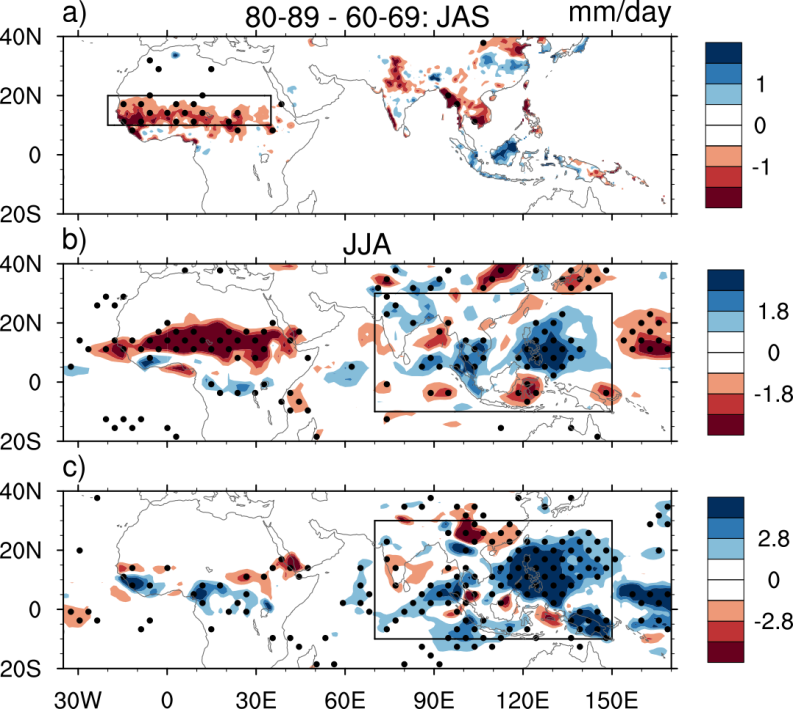


**Supplementary Figure S2 | Differences in JAS Sahel rainfall and JJA SAWPAM precipitation.** Differences in (a) JAS Sahel rainfall in the PRECL precipitation data, (b) JJA SAWPAM precipitation in the NCEP reanalysis data, and (c) JJA SAWPAM precipitation in ERA-40 reanalysis data, between 1980-1989 and 1960-1969. The boxes define Sahel (a) and SAWPSM (b and c). The stippled areas denote the values significantly exceeding the 95% confidence level (Student’s t-test). This figure was generated by the NCAR Command Language (Version 6.3.0) [Software]. (2016). Boulder, Colorado: UCAR/NCAR/CISL/TDD. <http://dx.doi.org/10.5065/D6WD3XH5>.

**Supplementary Figure S3**

**
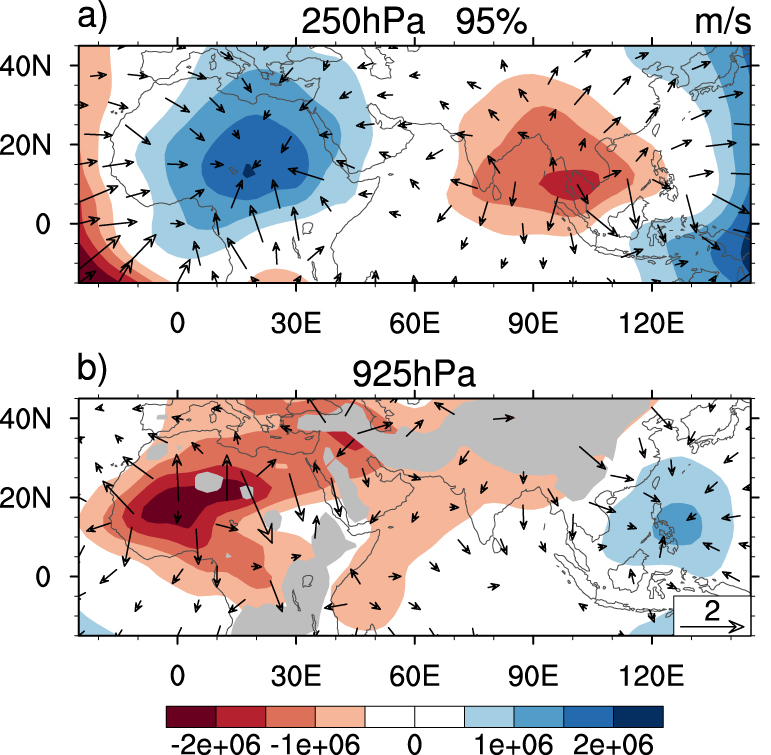
**

**Supplementary Figure S3 | Differences in JAS divergent winds.** Differences in JAS divergent winds (vector) and velocity potential (shaded) at (a) 250 hPa and (b) 925 hPa between 1980-1989 and 1960-1969. Only the vectors significantly at the 95% confidence level (Student’s t-test) are plotted. This figure was generated by the NCAR Command Language (Version 6.3.0) [Software]. (2016). Boulder, Colorado: UCAR/NCAR/CISL/TDD. <http://dx.doi.org/10.5065/D6WD3XH5>.

**Supplementary Figure S4**

**Supplementary Figure S4 | Differences in JAS SST.** Differences in global JAS SST between 1966 - 1981 and 1950 - 1965. The stippled areas denote the values that are statistically significant at the 99% confidence level according to the Student’s t-test. This figure was generated by the NCAR Command Language (Version 6.3.0) [Software]. (2016). Boulder, Colorado: UCAR/NCAR/CISL/TDD. <http://dx.doi.org/10.5065/D6WD3XH5>.

**Supplementary Figure S5**

**
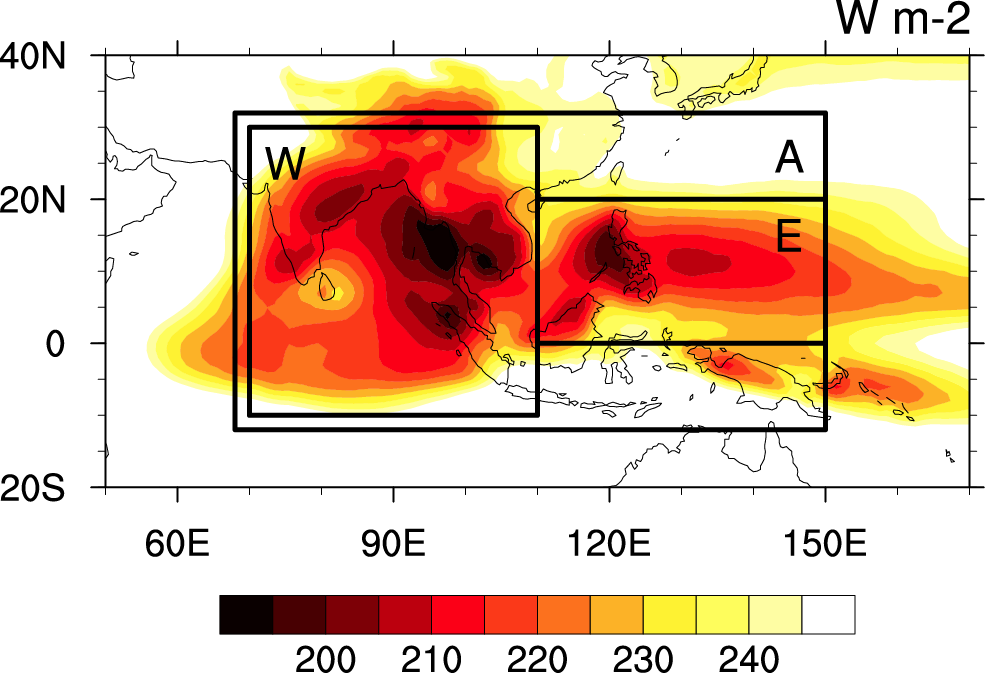
**

**Supplementary Figure S5 | Climatology of JAS outgoing longwave radiation.** Climatology of JAS outgoing longwave radiation from 1979 to 2010. The boxes define the regions (A, W, and E) of anomalous deep convectional latent heating for sensitivity experiments. This figure was generated by the NCAR Command Language (Version 6.3.0) [Software]. (2016). Boulder, Colorado: UCAR/NCAR/CISL/TDD. <http://dx.doi.org/10.5065/D6WD3XH5>.

**Supplementary Figure S6**


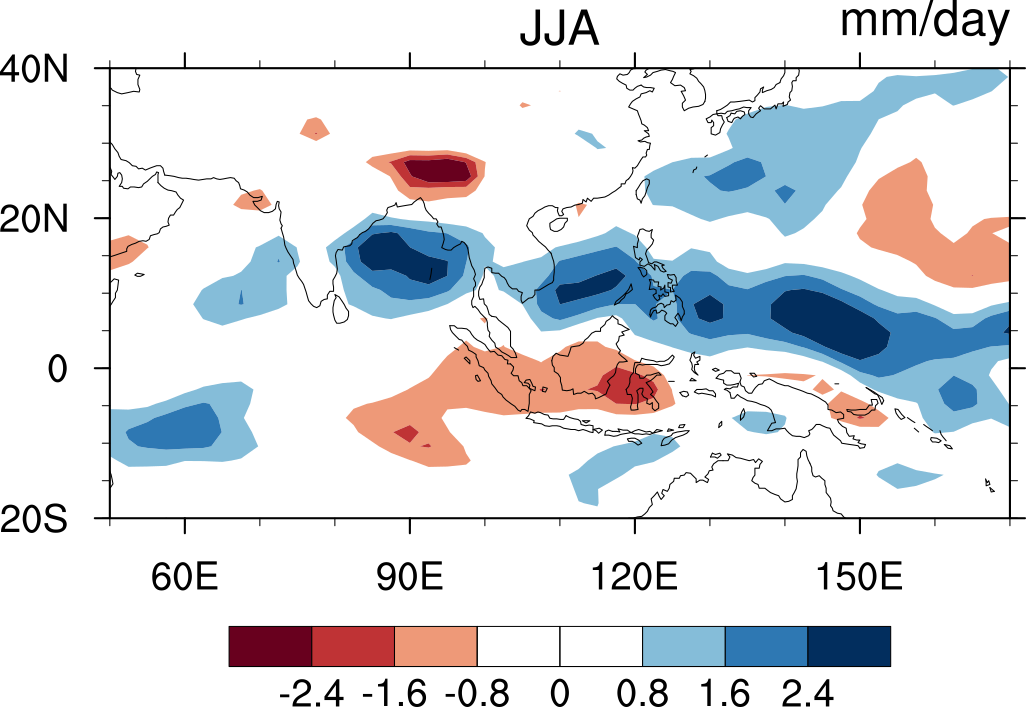


**Supplementary Figure S6 | Differences in JJA precipitation.** Differences in JJA precipitation between CON_80 and CON_60. This figure was generated by the NCAR Command Language (Version 6.3.0) [Software]. (2016). Boulder, Colorado: UCAR/NCAR/CISL/TDD. <http://dx.doi.org/10.5065/D6WD3XH5>.
